# The Protective Role of TLR2 Mediates Impaired Autophagic Flux by Activating the mTOR Pathway During *Neospora caninum* Infection in Mice

**DOI:** 10.3389/fcimb.2021.788340

**Published:** 2021-11-26

**Authors:** Jielin Wang, Xiaocen Wang, Pengtao Gong, Fu Ren, Xin Li, Nan Zhang, Xu Zhang, Xichen Zhang, Jianhua Li

**Affiliations:** ^1^ Key Laboratory of Zoonosis Research, Ministry of Education, College of Veterinary Medicine, Jilin University, Changchun, China; ^2^ Graduate College, Jinzhou Medical University, Jinzhou, China; ^3^ Department of Anatomy, Shenyang Medical College, Shenyang, China

**Keywords:** *Neospora caninum*, autophagy, mTOR, TLR2, anti-infection, innate immune, parasite proliferation

## Abstract

Autophagy has been shown to play an essential role in defending against intracellular bacteria, viruses, and parasites. Mounting evidence suggests that autophagy plays different roles in the infection process of different pathogens. Until now, there has been no conclusive evidence regarding whether host autophagy is involved in *Neospora caninum* infection. In the current study, we first monitored the activation of autophagy by *N. caninum*, which occurred mainly in the early stages of infection, and examined the role of host autophagy in *N. caninum* infection. Here, we presented evidence that *N. caninum* induced an increase in autophagic vesicles with double-membrane structures in macrophages at the early stage of infection. LC3-II expression peaked and decreased as infection continued. However, the expression of P62/SQSTM1 showed significant accumulation within 12 h of infection, indicating that autophagic flux was blocked. A tandem fluorescence protein mCherry-GFP-LC3 construct was used to corroborate the impaired autophagic flux. Subsequently, we found that *N. caninum* infection induced the activation of the TLR2–AKT–mTOR pathways. Further investigation revealed that TLR2–mTOR, accompanied by the blockade of autophagic flux, was responsible for impaired autophagy but was not associated with AKT. *In vitro* and *in vivo*, *N. caninum* replication was strongly blocked by the kinase inhibitor 3-methyladenine (3-MA, autophagy inhibitor). In contrast, rapamycin (Rapa, an autophagy inducer) was able to promote intracellular proliferation and reduce the survival rate of *N. caninum*-infected mice. On the other hand, the accumulation of autophagosomes facilitated the proliferation of *N. caninum*. Collectively, our findings suggest that activation of host autophagy facilitates *N. caninum* replication and may counteract the innate immune response of the host. In short, inhibition of the early stages of autophagy could potentially be a strategy for neosporosis control.

## Introduction

*Neospora caninum* (*N. caninum*), an intracellular protozoan parasite, is closely related to *Toxoplasma gondii* and causes abortion and reduced milk production in cattle, leading to financial losses worldwide (Reichel et al., 2013; Horcajo et al., 2016). Dogs (Langoni et al., 2013), cats (Silaghi et al., 2014), goats (Unzaga et al., 2014), and wild animals (Lempp et al., 2017) are all targets of *N. caninum* infection. Additionally, evidence indicates that *N. caninum* infections have been detected in humans (Lobato et al., 2006; Duarte et al., 2020).

Innate immune cells, such as macrophages, play a crucial role in controlling the initial parasite replication and pathogenesis of neosporosis, as these cells contribute to the first line of defense against intracellular infection. Upon *N. caninum* infection, various pattern recognition receptors (PRRs) of innate immune cells are activated, thus inducing a series of immune responses in the host (Yarovinsky et al., 2005; Mineo et al., 2010; Beiting et al., 2014; Davoli-Ferreira et al., 2016; Mansilla et al., 2016; da Silva et al., 2017; Wang et al., 2017). NF-κB, MAPK, and JAK/STAT signal pathways have been shown associated with infection (Jin et al., 2017; Nishikawa et al., 2018; Sharma et al., 2018). They influence the adaptive immune response by secreting many effector molecules including cytokines (Boucher et al., 2018; Jimenez-Pelayo et al., 2019; Miranda et al., 2019), controlling the proliferation and infection of *N. caninum*.

Autophagy is a protective mechanism that has evolved in eukaryotic organisms in response to environmental stress, achieving physiological homeostasis and internal environmental homeostasis through the degradation of intracellular components. It is a catabolic process that mainly includes initiation and formation of the autophagosome, docking and fusion with lysosomes, and subsequent degradation and reuse (Feng et al., 2014), which in brief, is manifested by increased LC3-II and degradation of p62/SQSTM1. It plays a key role not only in the growth and development of the organism but also in physiological and pathological processes such as immune defense. Autophagy has been proven to make an important contribution in defending against infection by microbial pathogens, including viruses, bacteria, and parasitic protozoa (Gomes and Dikic, 2014; Tao and Drexler, 2020). According to the mechanism of autophagy, invading pathogens are transported to the lysosome for degradation and elimination. In contrast, pathogens can also induce or disrupt host autophagy to promote intracellular survival and increased proliferation and thus promote intracellular infection.

The host–parasite relationship contributes to controlling infection (Kaye and Scott, 2011; Mukhopadhyay et al., 2020; Su et al., 2020). In parasitic infections, the complex immune system, acquired as a result of evolution, provides the most effective defense mechanism for the organism, which induces different effects, such as protecting the host, benefiting proliferation, or killing the parasite, while some immune responses will be harmful to the host.

Previous studies have shown that the autophagy pathway is conserved and essential in parasite infection. The autophagy pathway has a crucial role in *Trypanosoma cruzi* invasion (Romano et al., 2009) and provides protection against infection in mice (Casassa et al., 2019). During infection, *T. gondii* triggers the autophagic pathway in host cells which is beneficial to parasite recovery of host cell nutrients (Wang et al., 2009). The classical autophagy of the host is impaired by activating mTOR in the early stages of *Leishmania* infection, but at later stages of infection, autophagy is activated, which facilitates the survival of the parasite (Thomas et al., 2018).

In this study, we first addressed the key role of autophagy in the proliferation of *N. caninum in vivo* and in the pathogenesis of neosporosis *in vitro*. Furthermore, we found that *N. caninum* infection impairs TLR2–mTOR-dependent autophagy. Modulating autophagy in infected cells contributes to *N. caninum* proliferation and the development of neosporosis, meaning that rapamycin promotes severe infection, while 3-methyladenine (3-MA) has the opposite effect. This study provides a basis for exploring the pathogenesis of neosporosis and offers a new entry point for the prevention and control of neosporosis.

## Materials and Methods

### Animals

C57BL/6 mice (female, 8–10 weeks old) were purchased from the Changsheng Experimental Animal Center (Changchun, China), and *TLR2^−/−^* mice were purchased from the Model Animal Research Center of Nanjing University (Nanjing, China). All mice were housed in the National Experimental Teaching Demonstration Center of Jilin University, the environment was free of specific pathogens, and food and water were sterilized for use.

### Parasites, Cells, and Plasmids

*Neospora caninum* (*N. caninum*, *Nc*-1 isolate) and GFP-*Nc* were propagated in Vero (African green monkey kidney) in RPMI medium supplemented with 2% heat-inactivated fetal bovine serum (FBS). Then, 3–4 days after infection, monolayers of cells were scraped to harvest the tachyzoites, and cell suspensions were passed through a 27-gauge needle to lyse any remaining intact host cells. After centrifugation (2,000×*g*, 5 min), the tachyzoites were purified by density-gradient centrifugation on Percoll (Cornelissen et al., 1981). The pellet was collected and washed twice (2,000×*g*, 5 min) in PBS (pH 7.2). Tachyzoite density was measured using a hemocytometer to clarify the amount of parasite in the infection experiments. WT and *TLR2^−/−^* mice were injected intraperitoneally with 3 ml of 5% thioglycolate medium (BD Biosciences, New Zealand, USA) for 4 days, and the mice were humanely euthanized (Rutkowski et al., 2007) and sterilized with 75% alcohol. The cells were flushed with cold PBS, and peritoneal macrophages (PMs) were collected as previously described (Malvezi et al., 2014). The PMs were cultured in RPMI supplemented with 10% heat-inactivated fetal bovine serum. The culture medium was replaced after at least 12 h. RAW264.7 cells (American Type Culture Collection, Manassas, VA, USA), a mouse macrophage cell line, were routinely cultured in RPMI-1640 with 10% heat-inactivated FBS. The tandem fluorescent monomeric red fluorescent protein mCherry-GFP-LC3 was maintained in the laboratory.

### Transmission Electron Microscopy

In this assay, PMs in complete medium for 3 h were used as a negative control, and the PMs were challenged with *N. caninum* tachyzoites [multiplicity of infection (MOI) of 1:1] for 3 h. Cell samples were washed with PBS three times and centrifuged at 1,000×*g* for 10 min. Cells were collected at the bottom of 1.5 ml Eppendorf tubes. The cell pellets were fixed with 2.5% glutaraldehyde in PBS overnight at 4°C, postfixed in 1% OsO_4_ for 2 h, dehydrated with a graded series of ethanol, and then embedded in epoxy resin. Then, ultrathin sections were prepared and stained with uranyl acetate and lead citrate as previously described (Risco et al., 2012). The examination of autophagosome-like vesicles was performed by transmission electron microscopy (TEM) (HITACHI, Japan).

### Immunofluorescence

Confocal fluorescence microscopy was utilized to detect the expression of P62/SQSTM1 and the subcellular localization of NF-κB p65 in *N. caninum*-infected cells and measure the autophagic flux by mCherry-GFP-LC3. Cells were seeded in 22.1 mm dishes with coverslips. After infection, the coverslips were then washed three times with PBS, permeabilized with 0.25% Triton X-100 in PBS for 10 min, washed, and blocked in 3% BSA/PBS for 2 h at RT. After blocking, the samples were incubated with a 1:100 dilution of the antibodies overnight at 4°C, then washed and incubated with the suitable secondary antibody for 1 h at RT. The coverslips were stained with DAPI (Thermo Scientific) for 10 min before analysis on an Olympus FV1000 laser scanning confocal microscope (Japan). RAW264.7 cells were transfected with mCherry-GFP-LC3 when they grew to 60–70% confluence on coverslips, and after 24 h, they were infected with *N. caninum*. At 2 and 12 hpi, the cells were fixed and visualized by confocal microscopy.

### Western Blotting Analysis

The cells were washed in cold PBS and lysed with RIPA lysis buffer (Solarbio, R0020, Beijing, China) plus 1 mM phenylmethylsulfonyl fluoride (Boster, AR1178, Beijing, China) on ice. Protein concentrations were measured using the BCA Protein Assay Kit (Thermo Scientific, Waltham, MA, USA). Protein samples were separated on SDS–polyacrylamide gels (8% or 12%). Following the transfer to polyvinylidene difluoride membranes (PVDF), the protein-immobilized PVDF membranes were incubated overnight at 4°C with primary antibodies against LC3B (L7543, Sigma), p62/SQSTM1 (ab109012, Abcam), β-actin (60008-1, Proteintech), and GAPDH (ab181602, Abcam) and antibodies against Akt (#4691), phospho-Akt (Ser473) (#9271), mTOR (#2983), phospho-mTOR (Ser2448) (#2971), TLR2 (#13744), phospho-p65 (#3033S), and phospho-IκBα (#2859s) purchased from Cell Signaling Technology, Inc. (Danvers, MA, USA). After incubation with HRP-conjugated secondary antibodies for 1 h, the membranes were visualized by an enhanced chemiluminescence (ECL) Western Blot Detection System (Clinx Science Instruments, Co., Ltd., Shanghai, China). The TLR2/TLR1 agonist Pam3CSK4 (10 µg/ml, InvivoGen) was used to stimulate macrophages as a positive control to detect TLR2 expression.

### Stimulation and Experimental Design

To monitor the role of autophagy in the response to *N. caninum* infection in PMs, PMs were pretreated with rapamycin (AY-22989) (1 μM, S1039), 3-MA (10 mM, S2767), and bafilomycin A1 (Baf A1) (100 nM, S1413), which were purchased from Selleck Chemicals (Shanghai, China). To investigate the alteration of signaling pathways involved during *N. caninum* infection, PMs were pretreated with AKT inhibitor VIII (1.25 μM, S7776) and LY294002 (25 μM, S1105), which were purchased from Selleck Chemicals (Shanghai, China). The chemicals involved in the pretreatment experiments were removed prior to *N. caninum* stimulation, and the PMs were rinsed twice with sterile PBS.

Female C57BL/6 mice (8–10 weeks old) were randomized into seven groups (*n* = 8/each group), and 2 × 10^7^ *N. caninum* tachyzoites or GFP-*Nc* were infected by the intraperitoneal route: i) PBS group, mice received the same volume of PBS alone; ii) rapamycin group (Rapa), mice received rapamycin; iii) 3-MA, mice received 3-methyladenine; iv) *Nc*, *N. caninum* infected alone; v) rapamycin + *Nc* (Rapa + *Nc*), *N. caninum*-infected mice received rapamycin; vi) 3-MA + *Nc*, *N. caninum* mice received 3-methyladenine; and vii) *TLR2^−^* ^/^ *^−^* + *Nc*, *TLR2^−^* ^/^ *^−^* mice infected by *N. caninum*. Rapamycin [1 mg/kg/day (Zhao et al., 2017)] or 3-MA [15 mg/kg/day (Carmignac et al., 2011)] was injected intraperitoneally 1 day after infection by *N. caninum*, and the dose was given once a day for 7 or 30 days.

Peritoneal exudate cells were prepared by a peritoneal wash with 1 ml of ice-cold PBS. CD11b+ cells were magnetically labeled with APC-labeled anti-mouse/human CD11b (BioLegend). After washing, the cells were analyzed in a FACSAria flow cytometer (BD Biosciences). A minimum of 300,000 events were acquired per sample, and the collected data were analyzed in FlowJo version 10.0 (Tree Star Inc.).

### Assessment of Parasite Replication

Fluorescence microscopy observations and parasite-specific real-time quantitative PCR (qPCR) were employed to assess parasite replication.

PMs were challenged with *N. caninum* tachyzoites (MOI = parasite:cell; MOI = 1) for 24 h. When required, PMs were pretreated with Rapa and 3-MA for 2 h prior to *N. caninum* infection. At 24 h postinfection, samples were fixed in 4% paraformaldehyde for 20 min, permeabilized with PBS containing 0.25% Triton-X-100 for 10 min, blocked with PBS containing 3% bovine serum albumin (BSA) for 2 h, and washed three times for 5 min in PBS after each step. PMs were incubated with primary antibody against NcSAG1 (1:100) at 4°C overnight, washed three times with PBST, and then incubated with goat anti-rabbit fluorescein isothiocyanate (FITC)-conjugated secondary antibody (Proteintech) for 1 h at room temperature in the dark. F-actin and nuclei were stained with tetramethylrhodamine isothiocyanate (TRITC)-globulin (Yeasen, Shanghai, China) and 4′,6-diamidino-2-phenylindole (DAPI; Invitrogen, Carlsbad, CA, USA), respectively. The signals were detected using an Olympus FV1000 laser scanning confocal microscope (Japan). Infected cells were observed, and at least 100 parasitic vacuoles were counted to determine the number of parasites in each experimental sample.

Parasite DNA was analyzed by qPCR as described previously to monitor parasite replication in cells (Collantes-Fernandez et al., 2002). In brief, DNA of infected cells was extracted according to the instructions of the Genomic DNA Extraction Kit (TIANGEN, Beijing, China). Total DNA (500 ng) from the samples was used as the template for qPCR analysis using the FastStart Universal SYBR Green Master template. A pair of specific primers for the Nc5 sequence of *N. caninum* (forward: 5′-ACTGGAGGCACGCTGAAC-3′, reverse: 5′-AACAATGCTTCGCAAGAGGAA-3′) was used to amplify a 76-bp DNA fragment. The number of parasites was determined by a standard curve method using DNA isolated from *N. caninum*.

### Cell Viability Assay

Cell viability was measured by CCK-8 (Cell Counting Kit-8) after treatment. PMs were seeded at a density of 4 × 10^5^ cells/well in 96-well plates. After at least 12 h, the medium was changed, and the cells were treated with various reagents according to the experimental design. After treatment, 10 μl of CCK-8 reagent was added to 100 μl of medium in each well and incubated at 37°C for 1 h. The absorbance was measured at 450 nm.

### Statistical Analysis

Data are presented as the mean ± SEM. The significance of the variability between different treatment groups was analyzed by Student’s *t*-test and one- or two-way analysis of variance (ANOVA) using GraphPad Prism software (version 6.0). Significance is shown by *P < 0.05, **P < 0.01, and ***P < 0.001.

## Results

### Infection by *Neospora caninum* Enhances Autophagosome Formation in Macrophages

**Figure 1:**
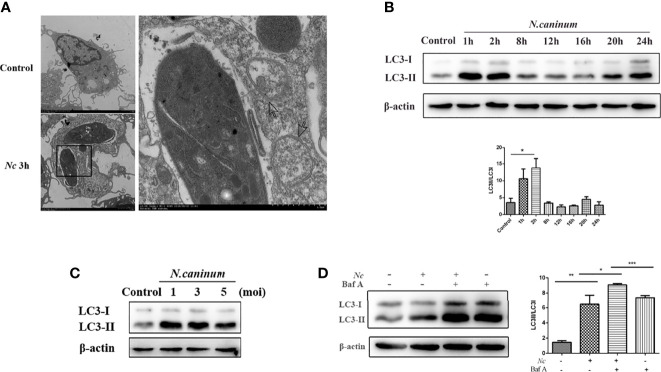
Autophagosomes accumulate in *Neospora caninum*-infected macrophages. **(A)** Representative transmission electron microscopy images of the control and *Nc*-infected mouse macrophages at 3 h postinfection. Arrows indicate representative autophagosomes. **(B)** Peritoneal macrophages were infected with *N. caninum* tachyzoites (MOI = 3), and total protein was extracted after 0, 1, 2, 4, 8, 12, 16, 20, and 24 h. The expression of LC3 and the ratio of LC3-II to LC3-I were examined at the indicated times. **(C)** Infection at different *N. caninum* infection ratios (MOI = 1, 3, 5) with macrophages for 2 h. Upregulated LC3 expression was observed in each group. **(D)** Peritoneal macrophages were pretreated with or without bafilomycin A1 (Baf A1; 100 nM) for 4 h prior to infection with *N. caninum* tachyzoites (MOI = 3), and the expression of LC3 was determined 2 h later. *P < 0.05, **P < 0.01, ***P < 0.001 compared with the control groups. The data shown are representative of three independent experiments.

To investigate whether autophagy could be involved during infection, peritoneal macrophages were infected with *N. caninum* and the autophagy level was determined. Electron microscopic examination indicated that compared with the control group, there were more vesicles with bilayer membrane structures containing organelles and cytoplasmic components that appeared in macrophages at 3 h postinfection (MOI = 3) (**Figure 1A**). LC3 is one of the signature proteins of autophagy, and it has been shown that autophagy can lead to increased expression of LC3 (Mizushima et al., 2010). In the current study, Western blotting was employed to observe the expression of LC3-II at different time points within 24 h after *N. caninum* infection. Consistent with the TEM results, the expression of LC3-II in macrophages was increased by infection, but LC3-II accumulation did not show time dependency, and the highest expression was observed after 2 h of infection (**Figure 1B**). Furthermore, macrophages were infected by *N. caninum* at different infection doses (MOI = 1, 3, 5), and the results showed a consistent upregulation of LC3 expression for all groups (**Figure 1C**). To further illustrate the activation of autophagy by *N. caninum* infection, Baf A, a late-autophagy inhibitor, was utilized. We focused on the early stages of infection, approximately 2 h postinfection. Western blot results indicated that, compared with the Baf A-treated group, the Baf A and *N. caninum* cotreatment group showed upregulated expression of LC3 (**Figure 1D**). All of these results suggest that infection by *N. caninum* in the early stages leads to an increase in autophagic structures in macrophages.

### Autophagic Flux Is Suppressed During *Neospora caninum* Infection

**Figure 2:**
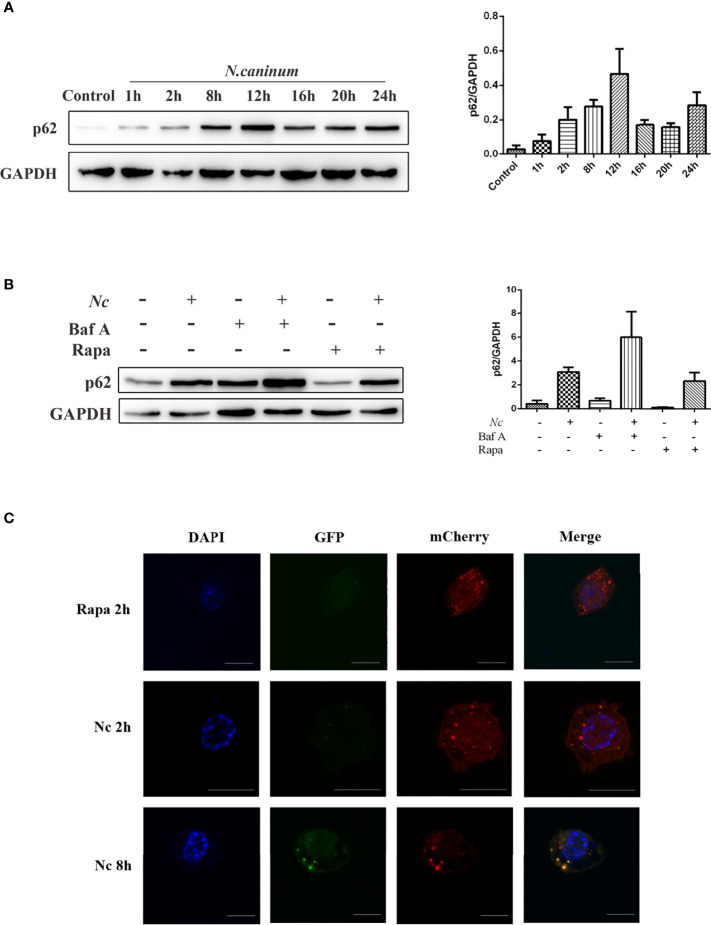
*Neospora caninum* infection suppresses autophagic flux. The changes in p62 were determined by Western blot analysis. **(A)** Peritoneal macrophages were infected with *N. caninum* tachyzoites (MOI = 3), and total protein was extracted after 0, 1, 2, 4, 8, 12, 16, 20, and 24 h, respectively. **(B)** Peritoneal macrophages were pretreated with rapamycin (1 μM) or bafilomycin A (100 nM) for 1 and 4 h and then infected with *N. caninum* tachyzoites (MOI = 3:1, parasite:cell). **(C)** RAW264.7 cells were transfected with mCherry-GFP-LC3, and cells treated with 1 μM rapamycin for 2 h were used as the positive control for the induction of autophagy. At 2 and 12 h postinfection, the cells were fixed and assessed for GFP and mCherry fluorescence. Scale bars: 10 μm. One of the three experiments conducted is shown.

The accumulation of autophagic structures caused by infection may be due to autophagy-induced or autophagy-prevented autophagic degradation (Mizushima et al., 2010; Klionsky et al., 2016); thus, the detection of autophagic flux is of critical importance (Zhang et al., 2013). SQSTM1, a cargo receptor protein, also known as p62, is a specific substrate for selective autophagy and an important component of the autophagosomal membrane. Western blotting was employed to examine the expression of p62/SQSTM1 at different time points within 24 h postinfection, and p62/SQSTM1 expression gradually increased, peaked at 12 h, and then decreased (**Figure 2A**). Furthermore, the cells were infected by *N. caninum* after Baf A and Rapa treatment, respectively. Rapa and *N. caninum* cotreatment stimulation decreased p62/SQSTM1 accumulation in macrophages compared with the *N. caninum*-infected group but displayed significantly higher expression than the Rapa group. In addition, pretreatment with Baf A resulted in more accumulation of *N. caninum*-induced p62/SQSTM1 compared with the *N. caninum*-infected group (**Figure 2B**). To test whether autophagic flux was blocked by infection, the tandem-tagged fluorescent reporter mCherry-GFP-LC3 was transfected into RAW264.7 cells and detected by fluorescence microscopy. The red signal of mCherry is responsible for demonstrating degradation, mainly because GFP fluorescence is less stable in the acidic environment of autophagic lysosomes and thus appears red. If colocalization appears yellow, it indicates impaired autophagic flux. The results showed that GFP puncta were increased by *N. caninum* at 12 h postinfection, and colocalization of red and green signals resulted in yellow puncta. However, the Rapa group showed red fluorescence because of the activated autophagy with complete autophagic flux. Taken together, these observations indicate that early infection by *N. caninum* can induce autophagy (**Figure 2C**). Autophagic flux was impaired with the *N. caninum* infection process.

### AKT–mTOR Signaling Is Activated in Macrophages During *N. caninum* Infection

**Figure 3:**
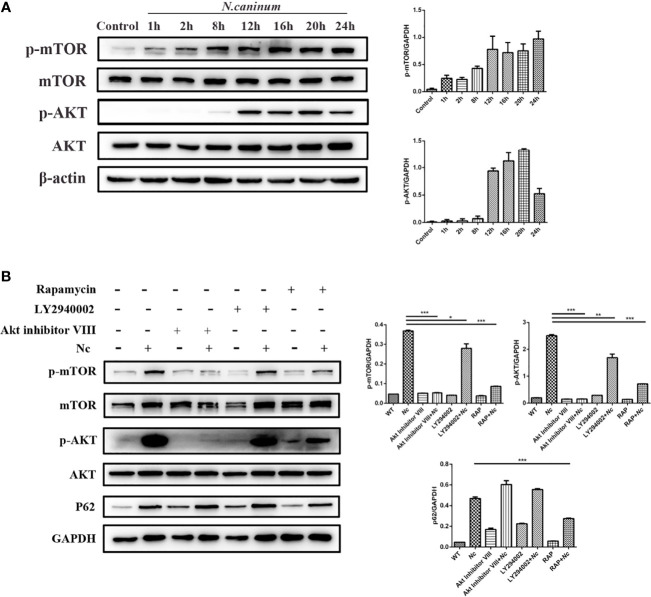
The effect of *N. caninum* infection on the AKT/mTOR signaling pathway in macrophages. **(A)** Peritoneal macrophages were infected with *N. caninum* tachyzoites (MOI = 3), and total protein was extracted at the indicated times. The ratios of p-mTOR, mTOR, p-AKT, AKT, and β-actin were detected by Western blot analysis. **(B)** The ratios of p62, p-mTOR, mTOR, p-AKT, AKT, and GAPDH were detected by Western blot analysis of cell lysates from peritoneal macrophages pretreated with or without rapamycin (1 μM), LY294002 (25 μM), or AKT inhibitor VIII (1.25 μM) for 1 h prior to infection with *N. caninum* tachyzoites (MOI = 3). The data shown are representative of three independent experiments. Bar graphs are expressed as the mean ± SEM, *P < 0.05; **P < 0.01; ***P < 0.001.

Autophagy is a complex physiological process, and a variety of signaling pathways are involved and contribute to the regulation of various processes of autophagy. The mTOR signaling pathway is important for regulating autophagy homeostasis. To characterize the effect of *N. caninum* infection on AKT and mTOR activation in macrophages, p-AKT and p-mTOR phosphorylation were examined using Western blotting. There was a significant time-dependent increase in the expression of p-AKT and p-mTOR in *N. caninum*-infected macrophages compared with the control group (**Figure 3A**). Combined with the previous experimental results (**Figures 1B**, **2A**), these results tentatively suggested that AKT–mTOR was possibly involved in *N. caninum*-induced inhibition of autophagy in the late stages of infection. For further validation, rapamycin (an inhibitor of mTOR), LY294002 (an inhibitor of PI3K), and Akt inhibitor VIII (an inhibitor of AKT) were used. The increased expression of p-mTOR and p-AKT induced by infection was significantly suppressed by coincubation with rapamycin (1 μM, 8 h). Not surprisingly, phosphorylation of mTOR was controlled by inhibition of PI3K and AKT (**Figure 3B**). Notably, rapamycin was the only treatment to reduce the expression of p62/SQSTM1 compared with the infection-only group (**Figure 3B**).

### TLR2 Deficiency Results in Attenuated p62/SQSTM1 Accumulation and Restores Autophagic Flux by Regulating mTOR Signaling Pathways in *Neospora caninum* Infection

**Figure 4:**
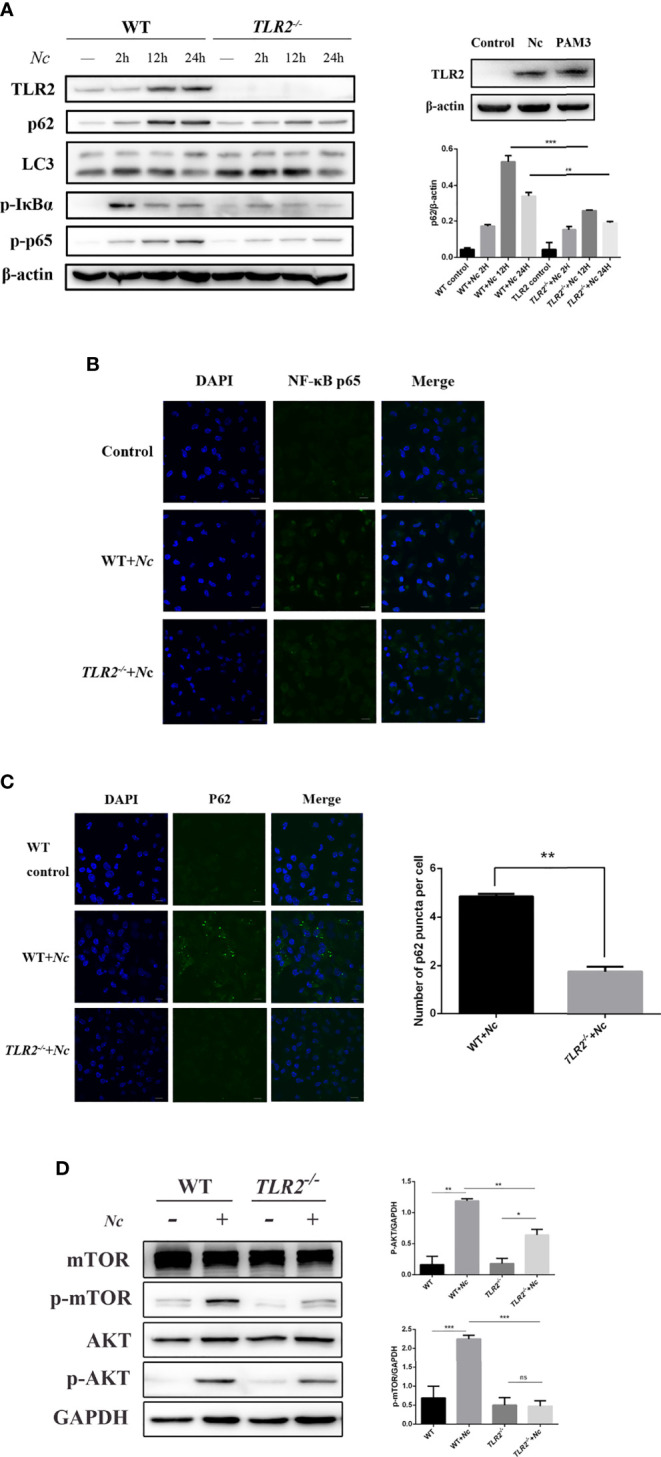
Activation of the TLR2–NF-κB and mTOR signaling pathways by *N. caninum* infection impairs autophagy in mouse peritoneal macrophages (PMs). **(A)** Western blot analysis of TLR2, p62, LC3, p-IκBα, p-p65, and β-actin in macrophages from WT and *TLR2****^−/−^*** mice infected with *N. caninum* (MOI = 3) for 2, 12, and 24 h. Macrophages were treated with Pam3CSK4 (10 μg/ml) as a positive control. The rate of p62/β-actin was shown. **(B)** Confocal microscopy was used to detect the translocation of NF-κB from the cytoplasm to the nucleus in both WT and *TLR2^−/−^* mouse peritoneal macrophages. Scale bars: 10 μm. **(C)** Eight hours postinfection with *N. caninum* (MOI = 3), the accumulation of p62/SQSTM1 was examined by confocal microscopy in both the WT and *TLR2^−/−^* groups. Quantitative analysis of the number of p62 puncta. The graph represents the average data of at least 100 cells in each experimental group in three independent experiments. Scale bars: 10 μm. **(D)** WT and *TLR2^−/−^* mouse PMs were infected with *N. caninum* (MOI = 3) for 8 h and then immunoblotted for whole-cell lysis analysis of p-mTOR and p-AKT protein expression. The phosphorylated mTOR and AKT amounts were quantified by densitometric analysis. The data are expressed as the mean ± SEM from three separate experiments. *P < 0.05; **P < 0.01; ***P < 0.001; ns indicates not significant.

Previous studies have demonstrated that TLR2 signaling is essential to protect the host against infection by *N. caninum* (Mineo et al., 2010). To investigate the role of TLR2 in the impairment of autophagic flux induced by *N. caninum*, WT and *TLR2^−/−^* PMs were used. Stimulation of PMs with *N. caninum* caused activation of TLR2–NF-κB signaling pathways including increased TLR2, p-p65, and p-IκBα (**Figure 4A**). In addition, the nuclear translocation of NF-κB p65 confirmed that the activation of NF-κB was dependent on TLR2 (**Figure 4B**). p62/SQSTM1 degradation was impaired after *N. caninum* infection, while mTOR, which negatively regulates autophagy, was activated. It was interesting to note that the expression of p62/SQSTM1 was reduced in response to *N. caninum* in *TLR2^−/−^* compared with the WT, but there was no reduction in LC3-II expression (**Figure 4A**). We next detected p62/SQSTM1 puncta in both the WT and *TLR2^−/−^* groups after *N. caninum* infection. Consistent with our Western blotting data, fewer p62/SQSTM1 puncta were observed in *TLR2^−/−^* PMs infected with *N. caninum* than in WT PMs (**Figure 4C**). In addition, we found a significant downregulation of p-mTOR and p-AKT expression in *TLR2^−/−^* mice compared with WT mice (**Figure 4D**). The results suggested that the AKT–mTOR signaling pathway triggered by *N. caninum* was activated through TLR2. Together with the previous results showing that inhibition of mTOR by rapamycin reduced p62/SQSTM1 expression but AKT and PI3K inhibitors did not (**Figure 3B**), we demonstrate that TLR2 is involved in the mTOR-dependent inhibition of autophagic flux, which is meaningful for studying the relationship between autophagy and innate immunity in *N. caninum* infection.

### TLR2 Deficiency Impairs Resistance to *N. caninum* Infection

**Figure 5:**
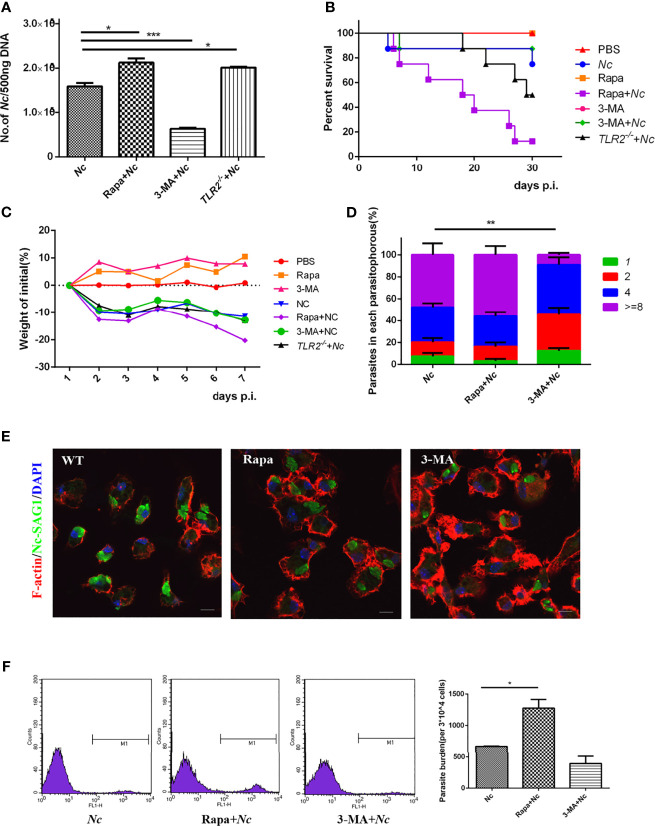
Roles of autophagy in host resistance to *N. caninum* infection. PMs were infected with *N. caninum* at MOI of 1 in the presence or absence of rapamycin (1 μM) or 3-methyladenine (3-MA) (2.5 mM) for 24 h. **(A)** The number of parasites in each group was detected by quantitative PCR. Female C57BL/6 mice were infected with 2 × 10^7^ *N. caninum* tachyzoites in the absence or presence of either Rapa or 3-MA and were monitored daily. **(B)** Survival of mice was monitored for 30 days. **(C)** Weight of mice was recorded daily within 7 days of infection. **(D)** Quantification of parasites in vacuoles in each group was monitored by fluorescence microscopy. **(E)** PMs were stained with polyclonal antisera against NcSAG1 and used to visualize *N. caninum*, and confocal microscopy was used to observe them. **(F)** Female C57BL/6 mice were intraperitoneally injected with 2 × 10^7^ of GFP-*Nc*; rapamycin (1 mg/kg/day) or 3-MA (15 mg/kg/day) was injected intraperitoneally 1 day after infection. Seven days later, peritoneal exudate cells were detected by flow cytometry. The data are expressed as the mean ± SEM from three separate experiments, *P < 0.05; **P < 0.01; ***P < 0.001.

Having observed that TLR2 is involved in the inhibition of autophagy in *N. caninum* infection, we decided to verify the anti-infection role of TLR2. The results showed that TLR2 deletion resulted in enhanced proliferation of *N. caninum* compared with infected WT cells (**Figure 5A**). Moreover, *TLR2^−/−^* mice were more susceptible to acute infection by *N. caninum* and showed increased mortality, but there were no obvious differences in weight loss between the *TLR2^−/−^* and WT groups (**Figures 5B, C** **)**. Our results were in accordance with previous studies (Mineo et al., 2010; Zhang et al., 2021), suggesting that TLR2 contributed to the proliferation and resistance to infection in *N. caninum*.

### The Proliferation of *Neospora caninum* and Host Resistance Correlate With Autophagy Alterations Induced by Autophagy-Regulating Reagents

To evaluate the role of autophagy in the restriction of *N. caninum* replication in macrophages, parasite number in macrophages was quantified. WT PMs were pretreated with Rapa or 3-MA followed by stimulation with *N. caninum*, and then the proliferation of *N. caninum* was observed in comparison to the infection-only group. An intracellular replication assay was performed to assess the proliferation efficiency of the parasite. Twenty-four hours after infection, the number of tachyzoites in the parasitophorous was counted by fluorescence microscopy. The results showed that both the *Nc* group and Rapa + *Nc* group displayed similar replication dynamics, but the 3-MA-pretreated group exhibited a slight decrease in parasite burden (**Figures 5A, D, E** **)**.

To further explore the role of autophagy in *N. caninum* infection *in vivo*, mice were randomly divided into the control group (PBS, Rapa, 3-MA, *n* = 8) and infection group (*Nc*, Rapa + *Nc*, 3-MA + *Nc*, *n* = 8). Each treatment group was given the corresponding therapy. Weight, survival time, and parasite burdens were monitored.

During infection, reduced body weight of mice was observed in all infected groups, compared with the initial body weight. There was more pronounced body weight loss in the Rapa + *Nc* group than in the other infection groups. The other two infection groups shared similar levels of weight loss (**Figure 5C**). In addition, survival rates were consistent with *in vitro* infection results, with autophagy induced by Rapa causing earlier disease exacerbation and significantly decreased survival rates (**Figure 5B**). Unexpectedly, the 3-MA + *Nc* group shared similar survival rates to those of the *Nc*-infected only group, despite 3-MA reducing *N. caninum* replication in *in vitro* experiments. To investigate the role of autophagy against *N. caninum* infection, GFP-*Nc* was injected intraperitoneally. Seven days postinfection, the percentage of *N. caninum*-infected CD11b+ cells was assayed by flow cytometry of extruding cells from the peritoneal cavity at the site of initial infection. Not surprisingly, the Rapa + *Nc* group exhibited the most severe infection, while 3-MA was found to reduce the rate of *N. caninum* infection, compared with the infected-only group (**Figure 5F**). The results suggest that modification of autophagy by autophagy regulators leads to changes in *N. caninum* proliferation and host resistance.

### Modulation of Autophagy Activity Does Not Affect Cell Viability

**Figure 6:**
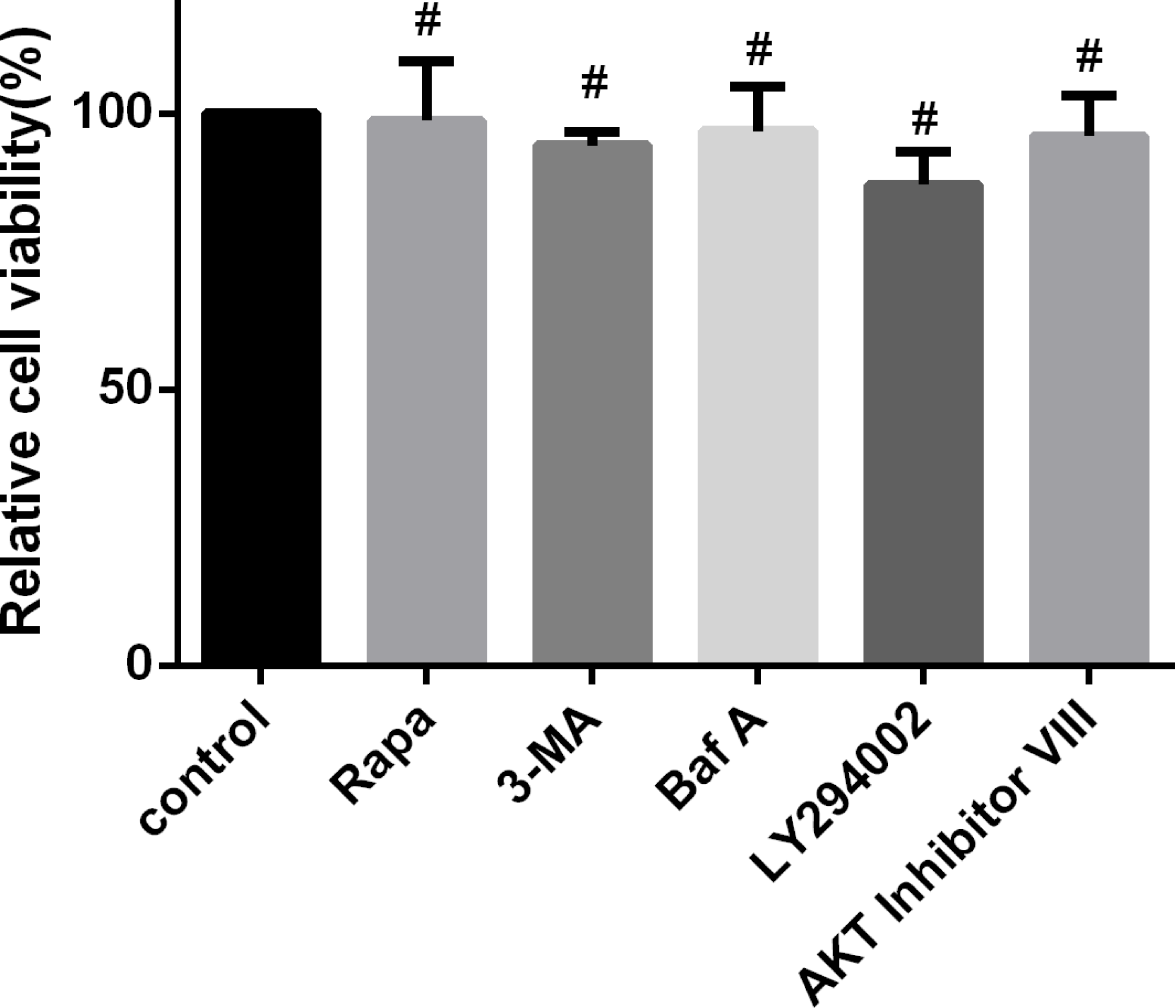
Regulation of autophagy and upstream signaling pathways does not affect cell viability. PMs were treated separately with rapamycin (1 μM), 3-MA (2.5 mM) for 24 h, Baf A (100 nM) for 4 h, LY294002 (25 μM), and AKT inhibitor VIII (1.25 μM) for 1 h. Cell viability was measured using a Cell Counting Kit-8 (CCK-8) assay. Data are shown as the mean ± SEM for three independent experiments. ^#^ *P* > 0.05.

In this study, to investigate the effect of autophagy during *N. caninum* infection, we altered autophagy with specific drugs, including Rapa, 3-MA, and Baf A. The relationships of signaling pathways and their activation during *N. caninum* infection were studied by altering signaling pathways with appropriate inhibitors, including AKT VIII and LY294002. We found no significant changes in cell viability by the CCK-8 assay, which provides a basis for further exploration of the relationship between autophagy and *N. caninum* (**Figure 6**).

## Discussion

Autophagy is a conserved cellular physiological process that plays a fundamental role in cellular, tissue, and physiological homeostasis through the lysosomal degradation pathway (Mizushima and Komatsu, 2011). In immune cells, autophagy also exhibits extraordinary immune functions in the fight against pathogenic microorganisms, in addition to its essential functions. Prior studies have noted the importance of autophagy in the process of infection by various microorganisms, such as bacteria, viruses, and parasites. Those include *Mycobacterium tuberculosis* (Gutierrez et al., 2004), *Listeria monocytogenes* (Py et al., 2007), and *Salmonella* (Nagy et al., 2019), whose intracellular proliferation is controlled by autophagy. Human immunodeficiency virus 1 (HIV-1) (Marin et al., 2003) and hepatitis C virus (Ait-Goughoulte et al., 2008) are also subject to autophagic degradation. However, the proliferation of protozoan parasites is affected by host cell autophagy, such as *T. gondii* (Wang et al., 2009) and *Leishmania* (Thomas et al., 2018).

Little to no research has attempted to assess the role played by autophagy in *N. caninum* infection. In this study, we demonstrated the accumulation of autophagosomes and increased levels of p62/SQSTM1 in *N. caninum*-infected cells. Additionally, failure of autophagosome–lysosome fusion was detected, which implied impaired autophagy. These results suggest that the activated autophagy mechanism triggered by *N. caninum* at the early stage of infection is incomplete, and autophagosomes do not efficiently fuse with lysosomes.

Various proteins are involved in the autophagy process. p62/SQSTM1, a well-studied autophagy receptor and substrate, interacts with Atg8 family members such as LC3 and ubiquitin and is conjugated to autophagosome membranes. This constitutes a mechanism of autophagic degradation for the delivery of selective autophagic cargo. The classical autophagic process includes the appearance of autophagosomes, fusion with lysosomes forming autolysosomes, and further degradation of autolysosomes, which, in brief, manifests as an increased LC3-II and the degradation of p62/SQSTM1. Moreover, p62/SQSTM1 performs other functions in addition to participating in autophagy. In particular, the interaction of p62/SQSTM1 with a number of signaling molecules increases its complexity as a reporter of autophagic flux. Even so, the detection of p62/SQSTM1 degradation is still an effective method that reflects the level of autophagic flux (Pankiv et al., 2007; Zheng et al., 2016). In the present work, with infection progression, we observed an accumulation of p62/SQSTM1 in macrophages within 12 h postinfection by *N. caninum*, which implied an impaired autophagic flux. In addition, we employed mCherry-GFP-LC3 to observe autophagic flux, which confirmed the impairment of autophagic flux presumed by p62/SQSTM1 accumulation. Of note, some other species were found to activate host autophagy, consistent with our current results. *Staphylococcus aureus*-infected bovine macrophages activate autophagy and increase LC3 expression at different times, accompanied by the accumulation of p62/SQSTM1 (Cai et al., 2020). Acting as a viral restriction factor, p62/SQSTM1 restricts dengue virus replication during infection (Metz et al., 2015). In addition, p62/SQSTM1 accumulates on PVs during *T. gondii* infection of human cells, and parasite growth is stunted in vacuoles positive for p62/SQSTM1 (Selleck et al., 2015). *N. caninum* triggered a large amount of p62/SQSTM1 in infected cells, and given this, we hypothesized that it should play a role in the anti-infection processes of *N. caninum*, which requires further investigation.

mTOR is considered a major negative regulator of autophagy, and the detailed mechanisms of its action have been intensively investigated (Jung et al., 2009; Kim et al., 2011). Few studies have focused on the effects of *N. caninum* infection on the host mTOR signaling pathway. A previous study showed that compared with *T. gondii* infection, the mTOR signaling pathway is not significantly enriched during *N. caninum* infection (Al-Bajalan et al., 2017). The inconsistent results are most likely due to distinct host cells, rather than macrophages, and a previous study targeted HFF cells.

The PI3K–Akt–mTOR pathway regulates a variety of cellular processes (Heras-Sandoval et al., 2014; Zabala-Letona et al., 2017), and autophagy is one of the most critical regulatory pathways, performing mainly negative regulation. Pretreatment of macrophages with a PI3K inhibitor (LY290042), AKT inhibitors (AKT VIII), and mTOR inhibitors (rapamycin) demonstrated that *N. caninum* infection activated the PI3K–Akt–mTOR signaling pathway. Contrary to expectations, this study did not find a significant difference in the inhibitor group in terms of p62/SQSTM1 expression, except for rapamycin, compared with the infection alone group. This possibly indicates that PI3K and AKT are not associated with *N. caninum*-induced inhibition of autophagic flux.

Although *N. caninum* activates AKT–mTOR and these signaling molecules have been reported to exert an inhibitory effect on autophagy, autophagosome formation was not blocked by *N. caninum* infection in macrophages. These findings prove that increased autophagosome structures are detectable in infected macrophages with increased levels of LC3-II in the early postinfection stage. It has been shown that *T. gondii* blocks autophagy through AKT signaling, but it has also been shown that autophagy can be utilized as an additional source of nutrition (Wang et al., 2009). Both starvation and drug-mediated (rapamycin and chloroquine) manipulation of autophagy influence the growth and survival of *Plasmodium* (Prado et al., 2015). The distinction between selective autophagy and canonical autophagy is believed to explain the opposite viewpoint. While parasites control selective autophagy to avoid elimination, canonical autophagy appears to be utilized by pathogens (Prado et al., 2015). The accumulation of autophagic vesicles due to autophagic flux damage is conducive to pathogen survival. As another example, autophagosomes are triggered by influenza A virus (IAV) but fail to form autophagolysosomes, which benefits the accumulation of viral elements (Liu et al., 2016). Treatment with 3-MA or knockdown of ATG5 and Beclin1 inhibits the early phases of autophagy, leading to impaired EBV replication (Granato et al., 2014). However, since the outbreak of autophagic vesicles occurs at the early stage of infection, and the first round of proliferation cannot be completed within a short period of time, it was not directly observed. It is only presumed that the formation of autophagosomes facilitates the growth of *N. caninum* in the early postinfection stage.

TLR2, a pattern recognition receptor, generally known as a sensor of bacterial lipoproteins, also senses molecular patterns from viruses and parasites (Oliveira-Nascimento et al., 2012). When TLR2 is activated, it recruits adaptor molecules to the intracellular Toll/interleukin-1 receptor (TIR) domain and ultimately activates NF-κB to regulate the expression of inflammation-associated genes (Oliveira-Nascimento et al., 2012). Previous studies found that TLR2 was upregulated when exposed to extracellular vesicles, soluble antigens, and glycosylphosphatidylinositols (GPIs) from *N. caninum* or parasite infection and regulated the secretion of a variety of cytokines (Mineo et al., 2010; Li et al., 2018; Debare et al., 2019). Whether TLR2 is engaged in the autophagy process during *N. caninum* infection in host cells has never been investigated. Our study showed that *N. caninum* infection increased the expression of the autophagic adaptor proteins p62/SQSTM1 and LC3-II in macrophages and triggered the TLR2–NF-κB signaling pathway. Studies on the relationship between p62/SQSTM1 and the NF-κB signaling pathway found that p62/SQSTM1 is involved in the regulation of NF-κB signaling (Pan et al., 2014); in turn, activation of NF-κB induces the expression of p62 (Yang et al., 2017), which forms a positive feedback loop. In this study, we observed a downregulation of p62/SQSTM1 expression in *TLR2^−/−^* PMs by *N. caninum* infection, accompanied by a downregulation of p-p65 expression and diminished p65 nuclear translocation. We speculate that the accumulation of p62/SQSTM1 in PMs by *N. caninum* may play a regulatory role in the activation of NF-κB, but the mechanisms of interaction need further investigation. Additionally, as a TLR2 ligand, the GPI anchor of *N. caninum* may also be involved in the autophagic process, which needs more supporting evidence.

To further elucidate the role of TLR2 in impaired autophagy by infection, we applied TLR2-deficient macrophages to observe the potential interaction between TLR2 and the AKT/mTOR pathway. AKT/mTOR expression was detected in both the WT and *TLR2^−/−^* groups. The results indicate that TLR2 deficiency downregulated both the expression and phosphorylation of AKT/mTOR. As previously mentioned, mTOR primarily plays a central negative regulatory role in autophagy. In our study, the phosphorylation of mTOR was positively correlated with TLR2, indicating an interaction between TLR2 and the mTOR pathway in the modulation of autophagy during *N. caninum* infection.

To gain insight into the role of autophagy in *N. caninum* infection, autophagy modulators were used to investigate the effect of autophagy on parasite proliferation as well as the resistance against infection. Autophagy inducers (rapamycin, mTOR inhibitor) and early autophagy inhibitors (3-MA, PI3K inhibitor) were employed. Parasite intracellular proliferation, percentage of infection at the initial location of the infection, and survival rate in the experimentally infected murine model were examined. Our data showed that *N.* caninum proliferation was promoted by rapamycin treatment in an autophagy-dependent manner and subsequently experienced rapid health deterioration and plummeting survival rates. Treatment with the early autophagy inhibitor 3-MA controlled the proliferation of *N. caninum*, although there was no significant difference in survival rate between the coadministration of the 3-MA group and the *N. caninum*-infected group, which may be attributable to the complex physiological environments. Similar findings have been observed in the study of many other pathogenic microorganisms, such as *T. cruzi* (Romano et al., 2009), *Plasmodium* (Thieleke-Matos et al., 2016), mouse hepatitis virus (Prentice et al., 2004), and duck Tembusu virus (Hu et al., 2020). The results indicate that the formation of early autophagosomes has a facilitative effect on *N. caninum* proliferation; in contrast, the activation of autophagy rather than exerting an anti-infection effect promotes *N. caninum* infection. These findings illustrate that modulation of host cell autophagy functions in the *in vivo* resistance to *N. caninum* infection, particularly in activating autophagy. Activated autophagy promotes the development of infection. Combined with infection-induced impairment of autophagy, we hypothesize that this is an anti-infective mechanism, although the early formation of autophagosomes is utilized by *N. caninum* to facilitate its proliferation.

In conclusion, we reported that the initial phase of autophagy triggered by *N. caninum* infection presented increased autophagosomes, especially in the early stages of infection. However, as the infection progressed, there was impaired autophagic flux. The autophagy involved in *N. caninum* infection is associated with the modulation of TLR2–mTOR signaling. Most unexpectedly, rapamycin, as an autophagic agonist, promoted the proliferation and infection of *N. caninum*. Taken together, the evidence suggests that *N. caninum* employs host autophagy machinery to facilitate proliferation and infection. Thus, the noted impairment of autophagic flux in macrophages triggered by *N. caninum* is beneficial to the resistance against *N. caninum* infection. Although the mechanism needs to be further elucidated in detail, our findings provide important evidence for understanding the role of autophagy in *N. caninum* infection and the underlying mechanisms.

## Data Availability Statement

The original contributions presented in the study are included in the article. Further inquiries can be directed to the corresponding authors.

## Ethics Statement

The animal study was reviewed and approved by the Animal Welfare and Research Ethics Committee at Jilin University.

## Author Contributions

JW, XW, PG, XCZ, and JL drafted the main manuscript and performed the data analysis. JW, PG, NZ, XZ, and XW planned and performed the experiments. JW, XCZ, and JL were responsible for the experimental design. JW, XL, NZ, XZ, FR, and JL were responsible for guiding and supporting the experiments and revising the manuscript. All authors contributed to the article and approved the submitted version.

## Funding

Project support was provided by the State Key Laboratory of Veterinary Etiological Biology, Lanzhou Veterinary Research Institute, Chinese Academy of Agricultural Sciences (grant no. SKLVEB2019KFKT006); National Natural Science Foundation of China (grant no. 31902296); and National Key Basic Research Program (973 program) of China (grant no. 2015CB150300).

## Conflict of Interest

The authors declare that the research was conducted in the absence of any commercial or financial relationships that could be construed as a potential conflict of interest.

## Publisher’s Note

All claims expressed in this article are solely those of the authors and do not necessarily represent those of their affiliated organizations, or those of the publisher, the editors and the reviewers. Any product that may be evaluated in this article, or claim that may be made by its manufacturer, is not guaranteed or endorsed by the publisher.
